# Potential antidepressant properties of aminophylline in male mice exposed to chronic restraint stress

**DOI:** 10.1007/s43440-026-00851-9

**Published:** 2026-04-07

**Authors:** Florence Ingred Samante, Christine Gale Galangue, Debbie Chenn Avelino, Antoneitte Joy Beltran, Michaela Rapada, Eldan Greg Pabia, Niel Andrew Bustamante, Chrislean Jun Botanas, Fred Lawrence Samante, Katrina Joy Bormate, Raly James Perez Custodio

**Affiliations:** 1 Department of Pharmacy, St. Scholastica’s College Tacloban Inc., Maharlika Highway, Brgy. Campetic, Palo, Leyte, 6501 Philippines; 2 Department of Psychiatry, University of Texas Southwestern Medical Center, Dallas, TX 75390 USA; 3 College of Medicine, University of the Philippines Manila, Ermita, Manila 1000 Philippines; 4 Networking Group Aging, Department of Ergonomics, Leibniz Research Centre for Working Environment and Human Factors at TU Dortmund (IfADo), Ardeystr. 67, 44139 Dortmund, Germany

**Keywords:** Aminophylline, Antidepressant, Behavior, Chronic restraint stress, Docking, Differentially expressed genes

## Abstract

**Background:**

Aminophylline, a bronchodilator used for treating airway obstruction, has been predicted through in silico models to exert antidepressant effects. However, there are still no studies that have validated these claims in a biological system. In this paper, we evaluated the antidepressant effects of aminophylline in mice subjected to chronic restraint stress (CRS).

**Methods:**

CRS was conducted for a duration of 15 days, with 4-hour daily stress exposure. Aminophylline (5 mg/kg, 10 mg/kg, and 20 mg/kg) and fluoxetine (10 mg/kg, positive control) were administered daily via intraperitoneal injection. Behavioral assessments, including the tail suspension test (TST), forced swimming test (FST), and sucrose splash test (SST), were conducted on days 0, 5, 10, and 15. Molecular docking and pathway analyses were performed to provide insight into its possible mechanism of action.

**Results:**

CRS exposure successfully induced depressive-like behavior, characterized by prolonged immobility in the TST and FST and diminished grooming activity in the SST. Administration of aminophylline attenuated these behavioral deficits, reducing immobility time and increasing grooming time in a dose-dependent manner. Molecular docking analysis demonstrated favorable binding of aminophylline to phosphodiesterase 3, phosphodiesterase 4, and the serotonin transporter, targets associated with antidepressant activity. Pathway analysis revealed upregulation of PPAR signaling, calcium signaling, and the synaptic vesicle cycle, while downregulating the glutamatergic synapse pathway.

**Conclusion:**

The study provides the first evidence of the antidepressant activity of aminophylline in a validated model of depression, prompting further clinical investigation into its therapeutic potential. Moreover, molecular and biochemical analyses are warranted to validate its precise mechanism of action.

**Supplementary Information:**

The online version contains supplementary material available at 10.1007/s43440-026-00851-9.

## Graphical abstract

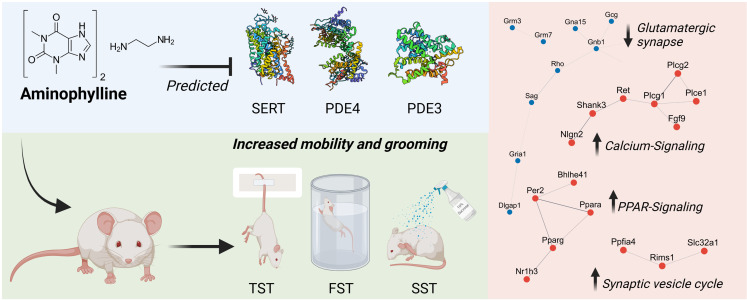

## Introduction

Major depressive disorder (MDD) is a condition characterized by persistent sadness, loss of pleasure, and diminished interest in daily tasks, which significantly impair one’s quality of life [1]. One of the well-established and extensively studied biological theories of depression is the monoaminergic theory, which suggests that imbalances in serotonin, norepinephrine, and dopamine contribute to depressive symptoms, thereby justifying the use of antidepressants [2]. However, the limited efficacy of these drugs highlights the possible involvement of other mechanisms integral to the development of MDD. Evidence of immune dysregulation and synaptic loss in patients with depression has directed attention toward neuroplasticity and neuroinflammatory pathways as potential drug targets [3]. This shift in focus is reinforced by clinical observations that current antidepressants fail to relieve symptoms in a substantial subset of patients (30%–50%), and approximately 30% develop treatment resistance [4, 5]. Such limitations underscore the urgent need for alternative treatments with novel mechanisms of action to improve clinical outcomes and deepen our understanding of the molecular basis of depression.

Drug repurposing provides a cost-effective approach to finding new drugs for the treatment of depression. By leveraging drugs with established safety profiles, this approach reduces the time and expenses associated with *de novo* drug discovery [6]. Aminophylline, a bronchodilator used for the management of asthma and airway obstruction, has emerged as a promising candidate for drug repurposing in the treatment of depression. This has been attributed to its combined pharmacological activities: adenosine receptor antagonism, phosphodiesterase inhibition, and anti-inflammatory activity.

Recent in silico analyses of genome-wide single-nucleotide polymorphism profiles have implicated the adenosine A1 receptor (ADORA1) as one of the genes conferring the highest risk for depression. The study proposed that antagonism of this receptor may confer an antidepressant effect, a mechanism known to be exerted by aminophylline [7]. Aside from blockade of adenosine receptors, aminophylline also acts as a phosphodiesterase 4 inhibitor (PDE4). Inhibition of PDE4 enhances cAMP signaling, promotes hippocampal neurogenesis, exerts neuroprotective activity, and has been shown to improve depressive-like symptoms in mice [8, 9]. In addition, aminophylline also has potent anti-inflammatory activity. This is highly significant given that there is now increasing evidence linking neuroinflammation and immune dysfunction to the pathophysiology of depression and the development of treatment resistance [10, 11]. This postulated three-pronged mechanism highlights aminophylline’s drug repurposing potential for depression. However, despite its prospective utility, there is currently no direct evidence of aminophylline’s antidepressant effects in biological models, underscoring the urgent need for in vivo studies to validate these findings.

In this study, we evaluated the antidepressant activity of aminophylline in male albino C57BL/6J mice with depressive-like behavior induced by chronic restraint stress (CRS). The behavioral effects of aminophylline were assessed using the tail suspension test (TST), forced swimming test (FST), and the sucrose splash test (SST). The results showed that aminophylline possesses antidepressant activity, as evidenced by a dose-dependent reduction in immobility time in the TST and FST, along with an increase in grooming time in the SST. Molecular docking simulations revealed favorable binding of aminophylline to phosphodiesterase enzymes PDE3 and PDE4, and to the serotonin transporter, targets associated with an antidepressant effect. Furthermore, its active component, theophylline, has been shown to modulate several pathways implicated in depression, including upregulation of the PPAR signaling, calcium signaling, and synaptic vesicle cycle pathways, along with downregulation of the glutamatergic synapse pathway. By integrating behavioral and computational findings, this study identifies aminophylline as a biologically and mechanistically supported candidate for antidepressant repurposing, warranting further translational investigation.

## Materials and methods

### Animals and housing/experimental conditions

Thirty-six male albino C57BL/6J mice (7 weeks old, 20–25 g) were obtained from Visayas State University, Leyte, Philippines. All experiments were conducted at the animal facility of the Department of Health Schistosomiasis Research and Training Center. The mice were group-housed in standard cages under a 12-hour light/dark cycle, with an ambient temperature of 21 ± 1 °C and relative humidity of 60 ± 10%. To facilitate adaptation, the mice underwent a seven-day acclimatization period prior to the start of the experiments, during which they were regularly handled to habituate them to the experimenter and minimize handling-related stress.

All behavioral tests and experimental procedures were performed during the light phase of the circadian cycle. Standard pellets and distilled water were provided ad libitum, except during chronic restraint stress (CRS) exposure and behavioral testing. The study followed the ARRIVE guidelines [12] and NIH regulations for the care and use of laboratory animals [13]. The principles of Replacement, Reduction, and Refinement (3Rs) were strictly observed [14], and all efforts were made to minimize animal suffering throughout the study.

### Experimental group and design

**Fig. 1:**
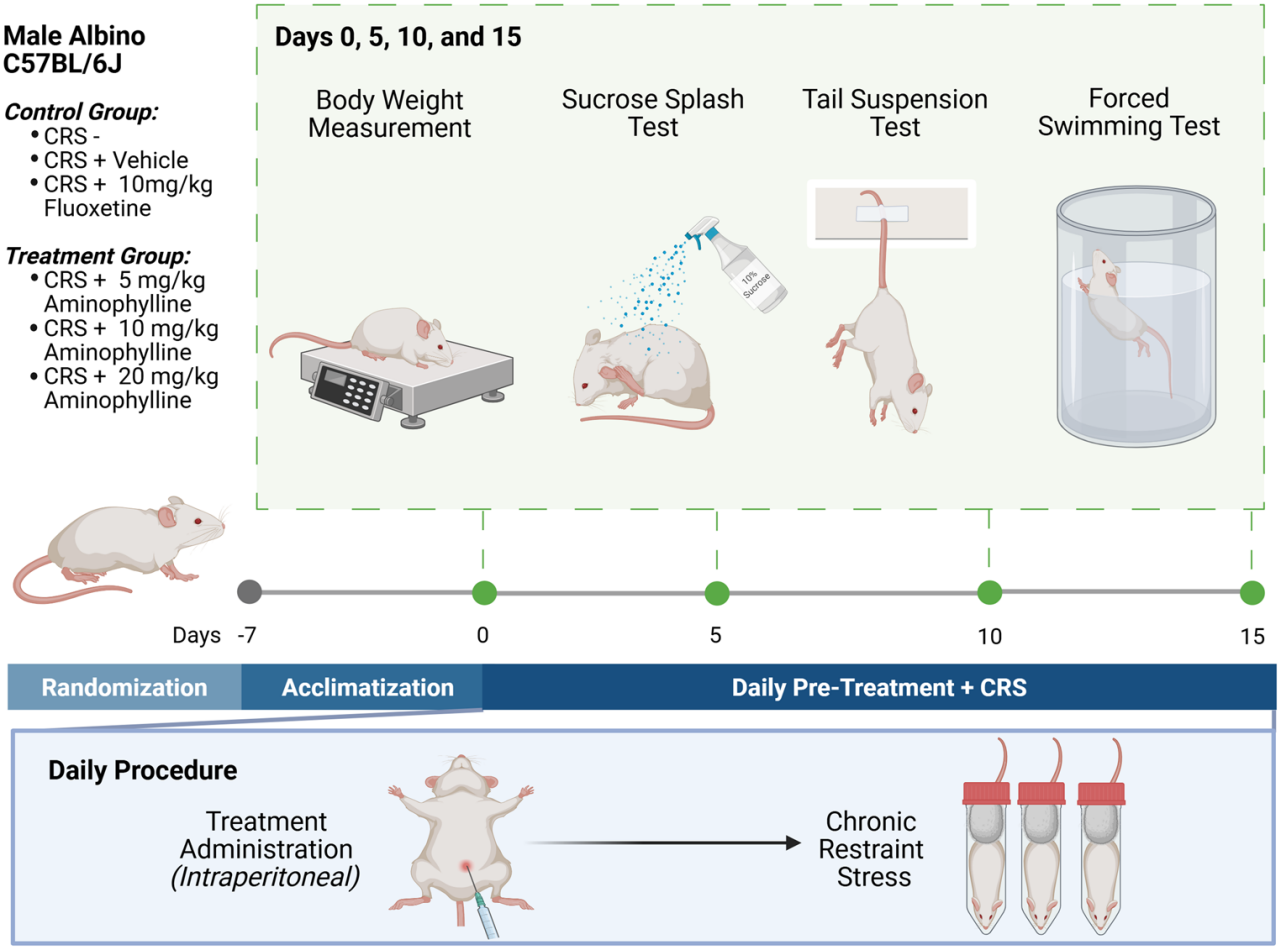
Experimental design. Male albino C57BL/6J mice were randomly assigned to aminophylline treatment groups (5, 10, or 20 mg/kg), a positive control group (fluoxetine), or a negative control group (vehicle). After administration of treatment, all animals were subjected to chronic restraint stress (CRS) (4 h/day) for 15 consecutive days. Body weight measurements and behavioral assessments were conducted on days 0, 5, 10, and 15. A non-stressed control group (CRS–) was included to provide baseline data and confirm the effects of CRS on depressive-like behavior. This figure was created with Biorender.com. Abbreviations: CRS, chronic restraint stress

Mice were randomly assigned to six groups (*n* = 6): CRS unexposed (CRS −), vehicle (CRS +), aminophylline 5 mg/kg, aminophylline 10 mg/kg, aminophylline 20 mg/kg, and the positive control, fluoxetine (Fig. 1). Following treatment administration, mice in the vehicle, aminophylline, and fluoxetine groups were subjected to CRS for four (4) hours daily for 15 consecutive days. Body weight measurements and behavioral assessments to evaluate depressive-like behavior, including the tail suspension test (TST), forced swimming test (FST), and sucrose splash test (SST), were performed at baseline before CRS (day 0) and on days 5, 10, and 15. Behavioral tests were administered in order of increasing stressfulness to minimize carryover effects on subsequent assessments, following the sequence of SST, TST, and FST [15]. A 120-minute recovery interval between tests was implemented to facilitate recovery from test-induced stress without compromising same-day evaluation of aminophylline’s pharmacological effects [16]. The sequence and timing of behavioral assessments were kept constant across all experimental groups. Prior to testing, mice were acclimated to the behavioral room for 60 min, and individual identifying marks were placed on their tails to ensure accurate tracking. At the conclusion of the experiment, all mice were humanely euthanized in accordance with ethical guidelines to ensure compliance with internationally accepted standards for animal welfare [12–14].

### Drug administration

In this study, sterile water for injection (vehicle) and fluoxetine were procured from Mercury Drug Corp. (Leyte, Philippines), while aminophylline (Aminosol^®^ ampule) was obtained from Eastern Visayas Medical Center (Leyte, Philippines). Fluoxetine (Prodin^®^), used as the positive control, was dissolved in the vehicle and administered at a dose of 10 mg/kg [17–19]. Aminophylline was administered at 5, 10, and 20 mg/kg, based on previously reported studies in rodents [20]. All drug treatments were delivered intraperitoneally (*ip*) 30 min before daily exposure to the CRS procedure [21, 22].

### Chronic restraint stress (CRS)

CRS was conducted following a previous protocol [23]. Mice were placed in a restraining device that substantially limited movement, with cotton balls used as stoppers to secure them. To minimize stress and encourage voluntary entry, the device was covered with a black towel, creating a dark environment conducive to entry. Each mouse underwent daily restraint for 4 h (8:00 AM to 12:00 PM) for 15 consecutive days to induce depressive-like behavior.

### Tail suspension test (TST)

TST is a widely used method for screening novel antidepressants. The test was conducted according to a previous protocol [24, 25] and is based on the principle that mice, when subjected to an inescapable stressor, exhibit immobility, which is indicative of a depressive-like state. For this experiment, a custom-built wooden box was used. Each mouse was suspended 50 cm above the ground by taping its tail approximately 1 cm from the tip using adhesive tape. The test lasted 6 min, during which the mice were recorded using a video camera. Immobility time was scored by a blinded experimenter during the final 4 min of the test. Immobility was defined as the mouse remaining completely motionless, passively hanging without any active movements.

### Forced swimming test (FST)

The FST is a well-established method for assessing depressive-like behavior in animal models. The test was conducted as described by Botanas et al. [24, 25], with minor modifications. Each mouse was placed in a 2-L glass beaker (19.3 cm in height, 13.1 cm in diameter) filled with water at 25 °C, with a depth of 10 cm, ensuring the mice could not touch the bottom or escape. The test lasted 6 min, during which the mice were recorded using a video camera. Immobility time was assessed during the final 4 min by a blinded experimenter. A mouse was considered immobile when it remained passively floating, making only minimal movements necessary to keep its head above water without active attempts to escape. After the test, mice were gently dried using paper towels to prevent hypothermia and then returned to their home cages for recovery. The water in each beaker was changed between sessions to maintain consistency across trials.

### Sucrose splash test (SST)

The SST is used to assess self-care and grooming behavior, which are often impaired in depressive-like states. Under normal conditions, mice engage in grooming behavior when their fur becomes soiled as part of their natural hygienic behavior [26]. A 10% sucrose solution was applied twice to the dorsal coat of each mouse using an atomizer spray. Following the application, mice were observed and recorded for 5 min in their respective testing cages. Total grooming time, including face washing, licking, and fur smoothing, was measured to assess self-care behavior [27].

### Molecular docking

Molecular docking is a computational technique used to predict the interaction between a ligand and a protein. The docking procedure was performed following the methods previously described [28] with some modifications. The 3D crystal structures of phosphodiesterase 3 (PDE3), phosphodiesterase 4 (PDE4), ADORA1, and serotonin transporter (SERT) (PDB entries [29–32]: 1SO2, 3HMV, 5N2S, and 5I73, respectively) were obtained from the Protein Data Bank (PDB) [33] (accessible at https://www.rcsb.org). The 3D structure of the ligand, aminophylline (PubChem CID: 9433), was retrieved from the PubChem database [34] (accessible at https://pubchem.ncbi.nlm.nih.gov/) in structure-data file (SDF) format.

#### Protein and ligand preparation

Protein and ligand structures were prepared using AutoDock Tools 1.5.7. Prior to docking, co-crystallized ligands, unwanted protein chains, and water molecules were removed from the protein structures. Missing protein residues were corrected, polar hydrogens and Kollman charges were added, and the processed protein files were saved in PDBQT format. The active sites of the target proteins were predicted using the COACH-D 2.0 web server [35], and the active site residues were mapped using AutoDock Tools 1.5.7 to accurately determine their location. A grid box was created to encompass the active site and its surrounding region. For ligand preparation, aminophylline was converted into PDBQT format after defining torsions.

#### Docking and visualization

Molecular docking was performed using AutoDock Vina [36], and the best pose was determined based on the highest binding energy rank. The docking results were further analyzed using PyMOL and BIOVIA Discovery Studio Visualizer (version 24.1.0.23298) to identify specific interactions between the drug and the target proteins. To ensure docking accuracy, redocking validation was performed by re-docking the co-crystallized ligands into the active sites of their respective proteins.

### Differential gene expression and pathway analysis

We used the microarray dataset GSE59895 (refer to https://www.ncbi.nlm.nih.gov/geo/query/acc.cgi?acc=GSE59895*)* to investigate the transcriptional perturbations induced by theophylline in brain tissue to explain its antidepressant effect. Raw data were retrieved from the Gene Expression Omnibus (GEO) database of the National Center for Biotechnology Information (NCBI; https://www.ncbi.nlm.nih.gov/geo/). Genes were annotated using the AnnotationDbi package with the org.Rn.eg.db database (R package version 3.19.1) [37]. Samples unrelated to the experimental design were excluded. Outlier genes were detected and removed using the goodSamplesGenes function in the WGCNA package [38]. Differential gene expression analysis was performed using limma [39], with a false discovery rate (FDR) threshold of 0.05, adjusted via the Benjamini–Hochberg correction. The resulting lists of differentially expressed genes (DEGs) were analyzed for physical subnetworks using STRING (version 2.2.0) [40] integrated into Cytoscape (version 3.10.4) [41], applying a confidence cutoff of 0.40. Large networks were represented by the top 10 core genes identified by the cytoHubba plugin (version 0.1) [42] using the maximal clique centrality method. Pathway enrichment for each subnetwork was performed using g: Profiler [43], focusing on Kyoto Encyclopedia of Genes and Genomes (KEGG) pathways containing 10–500 genes and applying a g: SCS threshold of 0.05. For each subnetwork, the KEGG pathway with the highest statistical significance was selected to represent its functional relevance.

### Statistical analysis

Statistical analyses were performed using the R programming language (version 4.5.1) [44]. Data normality was assessed using the Shapiro–Wilk test, and homogeneity of variance was verified using the Brown–Forsythe test. For parametric data, one-way analysis of variance (ANOVA) was applied to evaluate differences in immobility time (TST and FST), grooming time, and body weight among treatment groups on day 15, while two-way repeated-measures ANOVA with Geisser–Greenhouse correction was used to assess changes across treatment groups over time and relative to baseline. The Kruskal–Wallis test by ranks was used as an alternative for non-parametric data. When significant main effects or interactions were observed, Tukey’s honestly significant difference (HSD) test or Dunn’s multiple comparison test was performed for post hoc analysis. Data visualization was generated using BioRender and ggplot2 package [45], and results are expressed as mean ± standard error of the mean (SEM). Statistical significance was set at *p* < 0.05.

## Results

### Aminophylline attenuates CRS-induced depressive-like behavior in TST

**Fig. 2:**
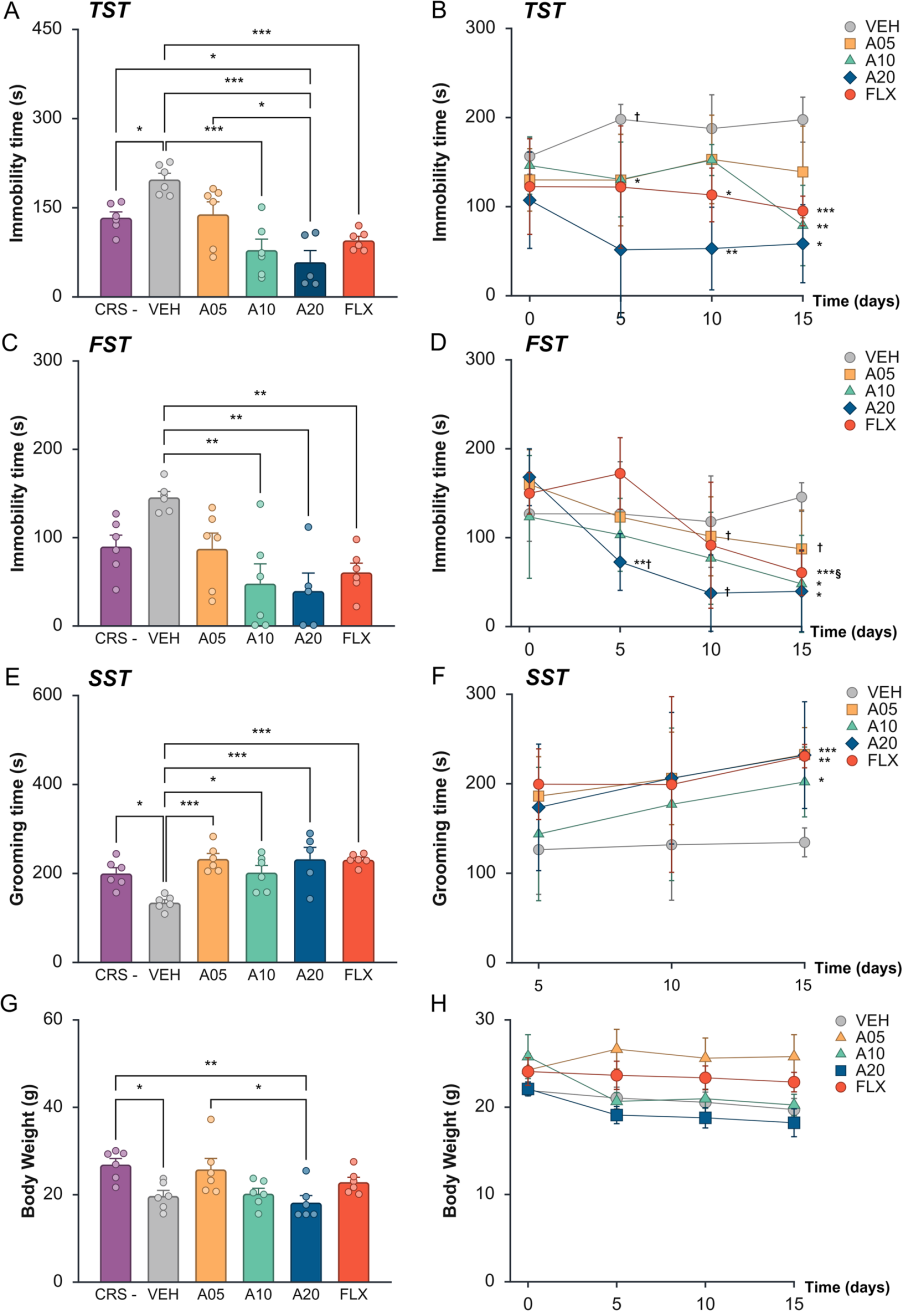
Aminophylline attenuates depressive-like behavior induced by CRS in TST, FST, and SST. (**A**) Immobility time of mice in TST after 15 days of exposure to CRS. (**B**) Temporal changes in behavior of mice in TST. (**C**) Immobility time of mice in FST after 15 days of exposure to CRS. (**D**) Temporal changes in behavior of mice in FST. (**E**) Grooming time in SST after 15 days of exposure to CRS. (**F**) Temporal changes in behavior of mice in SST. (**G**) Body weight measurement on day 15. (**H**) Body weight across the experimental period. Data are presented as mean ± SEM (*n* = 6 mice per group). Statistical analyses were conducted using one-way ANOVA followed by Tukey’s HSD post hoc test for panels (**A**), (**C**), (**E**), and (**G**), and two-way ANOVA followed by Tukey’s HSD post hoc test for panels (**B**), (**D**), (**F**), and (**H**). Comparisons were made versus the vehicle group (*p ≤ 0.05, **p ≤ 0.01, ***p ≤ 0.001) or versus baseline (†*p* ≤ 0.05, ‡*p* ≤ 0.01, §*p* ≤ 0.001). Abbreviations: A05, aminophylline 5 mg/kg; A10, aminophylline 10 mg/kg; A20, aminophylline 20 mg/kg; CRS, chronic restraint stress; CRS–, chronic restraint stress-unexposed group; FLX, fluoxetine; FST, forced swimming test; SEM, standard error of the mean; SST, sucrose splash test; TST, tail suspension test; VEH, vehicle

The TST was used to assess behavioral despair in mice, a core feature of depression (Fig. 2A; F_5,29_ = 10.888, *p* < 0.001). Mice subjected to CRS exhibited a significant increase in immobility time after 15 days of exposure compared to the unstressed controls (CRS+ vehicle vs. CRS–; *p* = 0.049), indicating successful induction of a depressive-like phenotype. Treatment with aminophylline at doses of 10 mg/kg (*p* < 0.001) and 20 mg/kg (*p* < 0.001) significantly reduced immobility time relative to the CRS+ vehicle group, effectively reversing the CRS-induced depressive-like behavior. Immobility times in aminophylline-treated mice were comparable to those of unstressed controls at 10 mg/kg (*p* = 0.132) and significantly lower at 20 mg/kg (*p* = 0.022). Mean immobility times for aminophylline (10 mg/kg: 78.8 s; 20 mg/kg: 58.4 s) were lower than those observed with fluoxetine (95.2 s), although the differences did not reach statistical significance.

Time-course analysis (Fig. 2B) revealed significant main effects of treatment (F_4,24_ = 11.05, *p* < 0.001) and a significant interaction (F_12,72_ = 2.370, *p* = 0.01), but no overall effect of time (F_2.51,60.29_ = 1.201, *p* = 0.31, Greenhouse–Geisser corrected). Post hoc analysis using Tukey’s multiple comparison test showed that the 10 mg/kg dose (*p* = 0.05) of aminophylline produced the earliest significant reduction in immobility relative to the vehicle, while the 20 mg/kg dose demonstrated sustained reductions starting at day 10 (*p* = 0.006) up to day 15 (*p* = 0.01). Relative to baseline, the aminophylline and fluoxetine treatment groups showed no significant change in immobility time after 15 days of exposure. However, distinct temporal patterns can be discerned among the aminophylline-treated cohorts. Mice receiving the 5 mg/kg dose displayed stable immobility times throughout the 15-day period, whereas those treated with the 10 mg/kg dose showed a gradual reduction in immobility time. At the highest dose, mice receiving 20 mg/kg exhibited a rapid decline in immobility time, achieving maximal reduction by day 5 and maintaining this effect thereafter.

### Aminophylline attenuates CRS-induced depressive-like behavior in FST

In parallel with the TST, the FST was employed as an additional measure to assess behavioral despair in mice (Fig. 2C; F_5,29_ = 5.955, *p* < 0.001). CRS exposure was associated with increased immobility time in the FST compared with the unstressed controls; however, this difference did not reach statistical significance (CRS+ vehicle vs. CRS–; *p* = 0.149). Consistent with TST findings, daily treatment with aminophylline at 10 mg/kg (*p* = 0.002) and 20 mg/kg (*p* = 0.001) significantly reduced immobility time following 15 days of CRS exposure relative to the CRS+ vehicle, suggestive of a similar behavioral reversal observed in the TST. Mice treated with aminophylline at these doses exhibited shorter immobility times than unstressed controls (10 mg/kg: *p* = 0.418; 20 mg/kg: *p* = 0.277) and the fluoxetine-treated group (10 mg/kg: *p* = 0.992; 20 mg/kg: *p* = 0.940), although these differences did not reach statistical significance.

Time-course analysis (Fig. 2D) revealed significant main effects of time (F_2.56,61.40_ = 15.29, *p* < 0.001, Greenhouse–Geisser corrected) and treatment (F_4,24_ = 4.043, *p* = 0.012), with a significant interaction (F_12,72_ = 2.543, *p* = 0.007). All aminophylline-treated groups exhibited a downward trend in immobility time from baseline, while the vehicle group remained unchanged. Relative to the vehicle, only the 20 mg/kg aminophylline group (*p* = 0.009) achieved a statistically significant reduction in immobility time prior to day 15. Relative to baseline, the 5 mg/kg dose produced a significant reduction starting on day 10 (*p* = 0.02), which was sustained until day 15 (*p* = 0.01). An earlier statistically significant reduction was observed for the 20 mg/kg group starting at day 5 and sustained until day 10. No statistically significant reduction was observed for the 10 mg/kg group throughout the observation period.

### Aminophylline improves depressive-like behavior in SST

Changes in grooming time in the SST serve as an indicator of depressive-like behavior, with reduced grooming reflecting impaired self-care commonly associated with depression (Fig. 2E; F_5,29_ = 7.329, *p* < 0.001). Mice subjected to 15 days of CRS exhibited a significant reduction in grooming time compared to the unstressed controls (CRS+ vehicle vs. CRS–; *p* = 0.025), confirming the induction of depressive-like behavior. Daily administration of aminophylline at all tested doses (5 mg/kg: *p* < 0.001; 10 mg/kg: *p* = 0.020; 20 mg/kg: *p* < 0.001) effectively reversed this deficit, producing a significant increase in grooming time relative to the CRS+ vehicle group. Grooming time in aminophylline-treated mice approached levels observed in unstressed controls (10 mg/kg: *p* = 0.999; 20 mg/kg: *p* = 0.637) and fluoxetine-treated animals (10 mg/kg: *p* = 0.685; 20 mg/kg: *p* = 0.999). Overall, these findings align with the effects observed in the TST and FST, supporting the antidepressant-like activity of aminophylline.

In the time-course analysis (Fig. 2F), two-way ANOVA revealed significant main effects of time (F_1.77,42.48_ = 3.745, *p* = 0.04, Greenhouse–Geisser corrected) and treatment (F_4,24 = 5.246_, *p* = 0.004), but no significant interaction (F_8,48_ = 0.249, *p* = 0.98). All aminophylline-treated groups exhibited a progressive increase in grooming time from day 5, whereas the vehicle group remained unchanged. By day 15, aminophylline at doses of 5 mg/kg (*p* < 0.001) and 10 mg/kg (*p* = 0.04) produced a statistically significant increase in grooming time relative to the vehicle.

### Treatment with aminophylline did not affect the mice’s body weight at low doses

Depression is often presented with physiological manifestations, among which is change in body weight (Fig. 2G; F_5,30_ = 4.840, *p* = 0.002). Fifteen days of CRS exposure induced significant weight loss compared to unstressed controls (CRS+ vehicle vs. CRS–: *p* = 0.036), indicating CRS-induced physiological stress. Aminophylline treatment at low doses did not significantly alter body weight relative to CRS+ vehicle (5 mg/kg: *p* = 0.106; 10 mg/kg: *p* = 0.999; 20 mg/kg: *p* = 0.984) or unstressed controls (5 mg/kg: *p* = 0.996; 10 mg/kg: *p* = 0.061). However, at 20 mg/kg, aminophylline caused a significant weight reduction compared to unstressed controls (*p* = 0.007), suggesting a potential weight-lowering effect at higher doses.

In the time-course analysis (Fig. 2H), two-way ANOVA revealed significant main effects of time (F_1.14,28.53_ = 5.342, *p* = 0.02, Greenhouse–Geisser corrected) and treatment (F_4,25_ = 3.109, *p* = 0.03), with a significant interaction (F_12,75_ = 2.023, *p* = 0.03). However, post hoc analysis revealed no significant changes in body weight within any treatment group over time relative to baseline or between groups at any time point compared to the vehicle.

### Molecular docking simulations of aminophylline-receptor interactions

To gain deeper insight into the antidepressant activity of aminophylline, molecular docking simulations were performed on selected enzymes and receptors that were postulated as key targets through which aminophylline exerts its antidepressant effect.

**Fig. 3:**
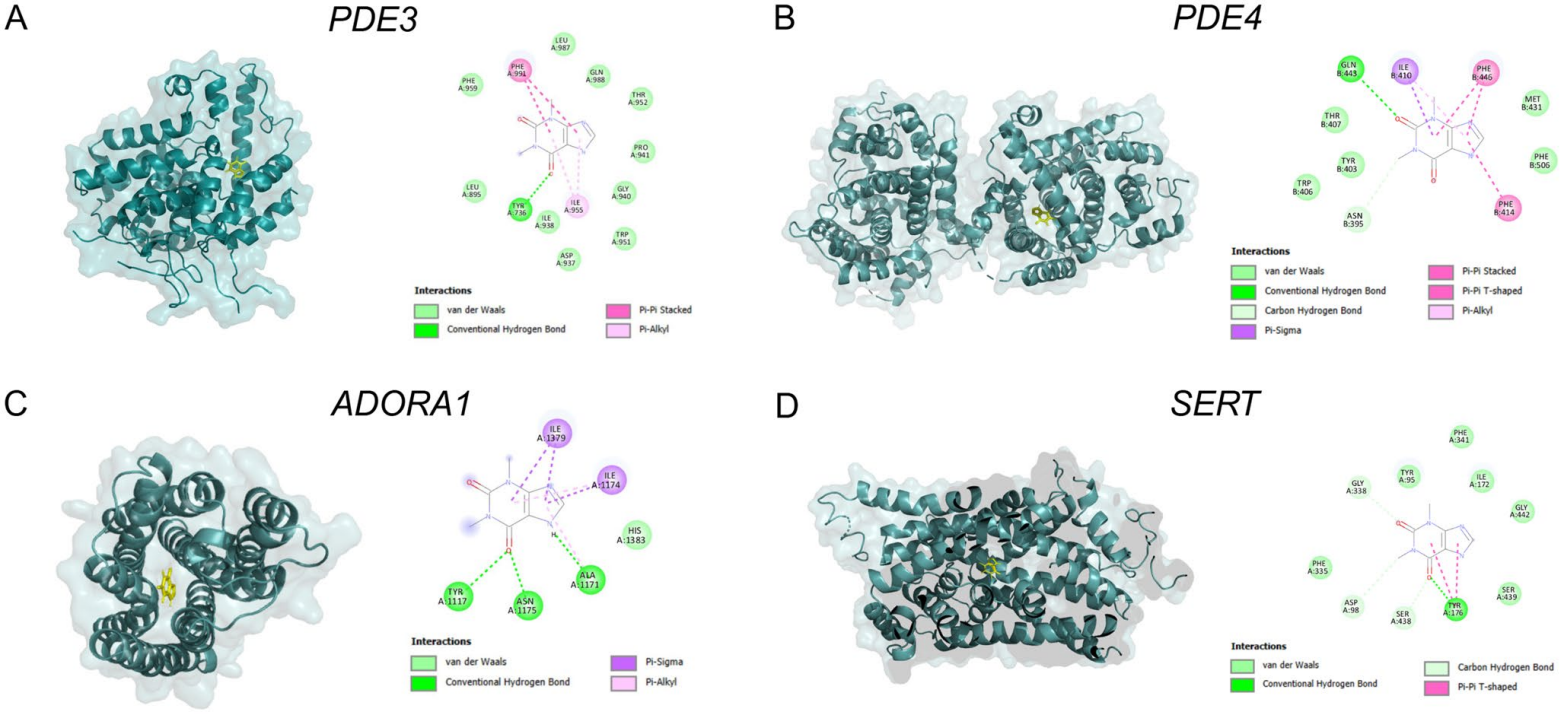
Predicted aminophylline-receptor interactions using molecular docking. (**A**) Docked structure of aminophylline with PDE3. (**B**) Docked structure of aminophylline with PDE4. (**C**) Docked structure of aminophylline with ADORA1. (**D**) Docked structure of aminophylline with SERT. Panels demonstrate the 3D binding conformations and the types of bonds formed at the ligand-binding pocket with key amino acid residues. Abbreviations: ADORA1, adenosine A1 receptor; PDE, phosphodiesterase; SERT, serotonin transporter

The first docking simulation was performed between aminophylline and the phosphodiesterase enzymes PDE3 and PDE4. These enzymes are established targets of aminophylline mediating its bronchodilator effect, and emerging evidence also implicates them in its potential antidepressant activity [46]. Docking with PDE3 and PDE4, therefore, served as the baseline for comparison with other aminophylline–receptor interactions while also providing insight into its possible mechanisms of antidepressant action. The docking simulation of aminophylline with PDE3 produced a binding energy of − 6.1 kcal/mol, characterized by van der Waals forces, conventional hydrogen bonds, pi–pi stacking, and pi–alkyl interactions (Fig. 3A). In comparison, docking with PDE4 yielded a stronger predicted affinity, with a binding energy of − 6.9 kcal/mol. The interaction profile included van der Waals forces, conventional hydrogen bonds, carbon–hydrogen bonds, pi–sigma interactions, pi–pi stacking, pi–pi T-shaped interactions, and pi–alkyl interactions (Fig. 3B).

In the second docking simulation, we evaluated the interaction of aminophylline with the adenosine A1 receptor (ADORA1). It has been postulated that inhibition of ADORA1 by aminophylline may contribute to its antidepressant effect [7]. The docking simulation revealed a relatively weaker binding affinity (*ΔG_bind* = − 5.0 kcal/mol) compared with the phosphodiesterase enzymes. The interaction profile included van der Waals forces, conventional hydrogen bonds, pi–sigma interactions, and pi–alkyl interactions (Fig. 3C).

Finally, the potential interaction of aminophylline with the serotonin transporter (SERT), a well-established target of many antidepressants, was also assessed. Docking results showed a binding energy of aminophylline (*ΔG_bind* = − 6.2 kcal/mol), comparable to that of its bronchodilatory targets, PDE3 and PDE4, although weaker than that observed with the positive control, fluoxetine bound to SERT (*ΔG_bind* = − 8.6 kcal/mol). Key interactions between aminophylline and SERT involved van der Waals forces, conventional hydrogen bonds, carbon–hydrogen bonds, and pi–pi T-shaped interactions (Fig. 3D).

Docking simulations revealed that aminophylline may interact with key targets implicated in depression, including the serotonin transporter, which is a principal target of antidepressants, and the phosphodiesterase enzymes PDE3 and PDE4, which have been increasingly associated with depression mechanisms. Although its binding affinity with the ADORA1 receptor was comparatively weak, the potential additive contribution of this interaction warrants consideration. These findings collectively suggest plausible molecular targets that may underlie the observed antidepressant activity of aminophylline and provide a basis for further experimental validation.

### Differential gene expression and pathway analysis

**Fig. 4:**
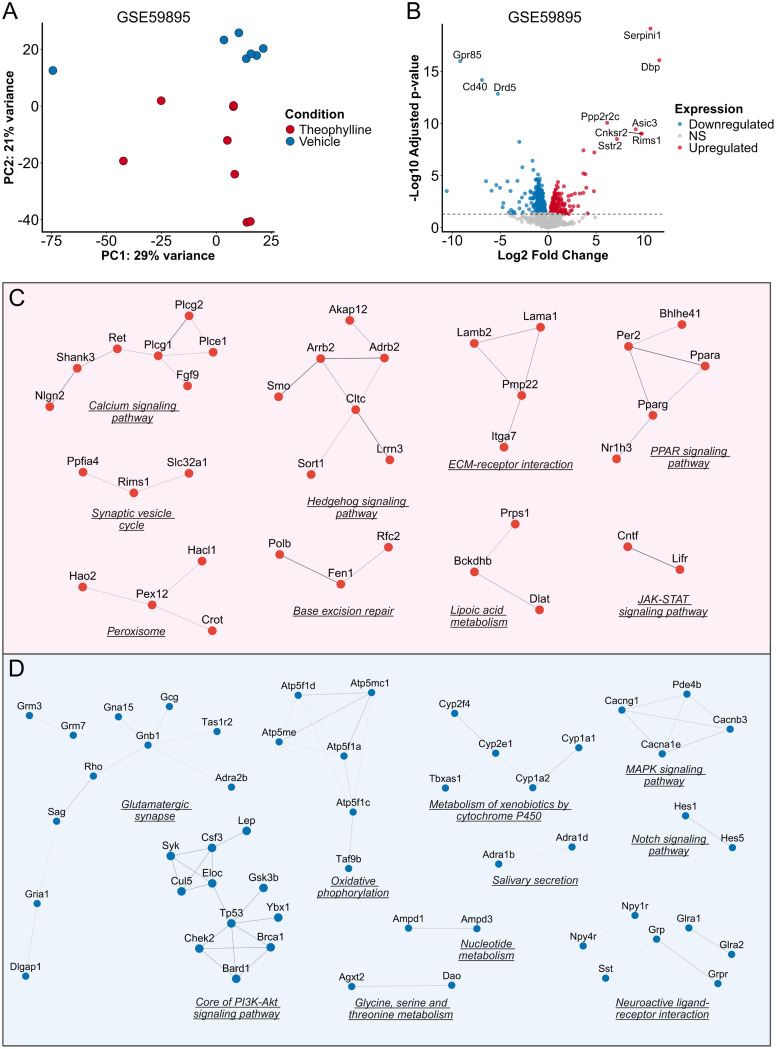
Theophylline, the active component of aminophylline, modulates key pathways in depression. (**A**) Principal component analysis of the theophylline- and vehicle-treated rat brain tissues in the GSE59895 microarray dataset. (**B**) The top ten differentially expressed genes. (**C**) Upregulated physical subnetworks annotated with the KEGG pathway presenting the highest statistical significance. (**D**) Downregulated physical subnetworks annotated with the KEGG pathway presenting the highest statistical significance. Abbreviations: KEGG, Kyoto Encyclopedia of Genes and Genomes

We analyzed the publicly available microarray dataset GSE59895 to further investigate the mechanism of the antidepressant effect of aminophylline. The dataset is comprised of seven rat brain tissues exposed to theophylline, the active component of aminophylline, together with seven control brain tissues. Principal component analysis (PCA) of the samples showed clear separation between theophylline-treated and control samples (Fig. 4A). The first two principal components accounted for 50% of the total variance (PC1: 29%, PC2: 21%). Theophylline-treated samples clustered distinctly in the lower half along PC2, while the control samples clustered in the upper right quadrant. This suggests that theophylline treatment induced substantial transcriptional changes compared with the vehicle.

Differential gene expression analysis revealed 221 upregulated genes and 392 downregulated genes (Supplementary Table). The top ten differentially expressed genes are labeled in Fig. 4B. The upregulated genes mapped into nine physical subnetworks identified by KEGG as the calcium signaling pathway, synaptic vesicle cycle, peroxisome, hedgehog signaling pathway, base excision repair, extracellular matrix (ECM)–receptor interactions, lipoic acid metabolism, peroxisome proliferator–activated receptor (PPAR) signaling pathway, and Janus kinase/signal transducers and activators of transcription (JAK–STAT) signaling pathway (Fig. 4C).

Meanwhile, the downregulated genes mapped into 13 networks corresponding to 10 distinct pathways identified by KEGG, namely the glutamatergic synapse, phosphatidylinositol 3-kinase (PI3K)/protein kinase B (AKT) signaling pathway, oxidative phosphorylation, glycine, serine, and threonine metabolism, nucleotide metabolism, salivary secretion, metabolism of xenobiotics by cytochrome P450, mitogen-activated protein kinase (MAPK) signaling pathway, notch signaling pathway, and neuroactive ligand–receptor interaction (Fig. 4D).

## Discussion

Depression is a complex and multifaceted disorder strongly influenced by emotional distress, environmental stressors, genetic predisposition, or a combination of these factors [47, 48]. Although antidepressants remain the primary treatment, their clinical utility is often limited by inadequate response, treatment resistance, delayed efficacy, and adverse effects. These challenges highlight the need for alternative therapeutic approaches. Drug repurposing offers an efficient strategy to address this gap by leveraging existing pharmacological agents while reducing the time and resources required for drug development. Aminophylline, a methylxanthine bronchodilator, has emerged as a promising candidate for repurposing in depression owing to its predicted multipronged mechanism of action targeting several pathways implicated in the disorder. To the best of our knowledge, the present study is the first to substantiate this claim with biological evidence of antidepressant efficacy in a validated animal model.

The present study utilized the CRS model, which is a widely recognized method for inducing depressive-like behavior in rodents [49]. We first validated this model by comparing the CRS-exposed group to the unexposed control. CRS successfully induced depressive-like behaviors in mice, as evidenced by increased immobility time in both the TST and FST, along with reduced grooming time in the SST. These findings are consistent with previous reports showing that prolonged exposure to stress impairs natural coping mechanisms, resulting in behavioral despair and diminished self-care [50, 51]. The efficacy of fluoxetine as the positive control, which significantly reversed CRS-induced behavioral alterations across all tests, further validates the experimental model. Notably, a lower dose of fluoxetine (10 mg/kg) was effective in ameliorating these behavioral deficits, suggesting that prolonged treatment duration may enhance therapeutic outcomes at reduced doses, potentially minimizing side effects [17, 53].

The antidepressant activity of aminophylline was evaluated using the same battery of behavioral tests, which provides a holistic assessment by capturing distinct yet interconnected dimensions of depressive-like states. Aminophylline exhibited a dose-dependent antidepressant effect in the tests that measure behavioral despair, specifically the TST and FST. Even at a 10 mg/kg dose, aminophylline significantly reduced immobility time in these tests, suggesting that low doses may suffice to counteract stress-induced behavioral deficits. The TST produced more homogeneous results than the FST and was more sensitive in detecting behavioral changes, which may account for the absence of a statistically significant difference in the FST between the CRS-exposed group and the control. While the TST and FST primarily evaluate behavioral despair, the SST addresses self-care deficits, offering insights into both passive and active coping mechanisms. The findings in the SST further reinforce the antidepressant effects observed in the TST and FST, showing that aminophylline effectively reverses stress-induced deficits in self-care behavior. This multifaceted approach strengthens the reliability of the findings and provides a more nuanced understanding of the depressive phenotype induced by chronic stress. The early onset of aminophylline’s antidepressant activity, observed by day 5, along with gradual improvements over time, suggests its potential as a fast-acting treatment for depression. This provides a significant advantage over conventional antidepressants, which are typically associated with delayed therapeutic onset. This rapid onset parallels advances seen with novel agents such as ketamine and its analogs, which exert fast-acting effects through glutamatergic and neuroplasticity-related pathways, and further justifies investigation into non-monoaminergic mechanisms of action for aminophylline [52].

Several plausible mechanisms may account for the observed antidepressant effects. Molecular docking revealed favorable binding affinity of aminophylline to ADORA1, PDE3, PDE4, and SERT. In the context of monoamine neurotransmission, the observed binding affinity to SERT suggests that aminophylline may modulate serotonin levels, akin to the mechanism of selective serotonin reuptake inhibitors (SSRIs) such as fluoxetine. Beyond monoamine modulation, differential gene expression and pathway analysis demonstrated that theophylline, the active constituent of aminophylline, upregulates calcium signaling and the synaptic vesicle cycle. Calcium influx triggers vesicle mobilization and exocytosis, releasing neurotransmitters into the synapse. Upregulation of the synaptic vesicle cycle then ensures that emptied vesicles are rapidly recycled, maintaining a continuous supply of neurotransmitters for ongoing transmission [53]. By modulating both steps of this process, aminophylline permits stronger and more sustained synaptic signaling. Additionally, theophylline was found to downregulate the glutamatergic synapse pathway, which is significant given that excessive glutamate receptor activation can produce oxidative stress and neuronal cell death [54]. Aminophylline’s capacity to simultaneously enhance monoaminergic neurotransmission while attenuating glutamate-induced excitotoxicity represents a mechanistically distinct and potentially advantageous pharmacological profile compared to conventional antidepressants.

Neuroinflammation represents an additional and increasingly recognized mechanism in the pathophysiology of depression, particularly in treatment-resistant cases [55, 56]. Aminophylline’s binding affinity to phosphodiesterase (PDE) isoforms PDE3 and PDE4, as revealed by molecular docking, suggests a capacity to attenuate the inflammatory response. Complementing this, pathway analysis demonstrated upregulation of PPAR signaling, which is also known to attenuate neuroinflammatory responses [8, 57]. By targeting neuroinflammation through both PDE inhibition and PPAR activation, aminophylline may offer a novel therapeutic option for patients who do not respond to conventional antidepressants. Furthermore, both PDE inhibition and PPAR signaling upregulation have been linked to increased expression of brain-derived neurotrophic factor (BDNF), a critical mediator of synaptic plasticity and neuronal survival [58, 59]. This mechanism may differ from traditional antidepressants, such as SSRIs, which primarily modulate serotonin levels without directly enhancing neurotrophic signaling. By promoting BDNF expression, aminophylline could facilitate more sustained synaptic remodeling and neuroprotection, potentially contributing to long-lasting antidepressant effects. It is also noteworthy that theophylline was found to downregulate the PI3K–AKT and MAPK signaling pathways, which have previously been noted to promote an antidepressant effect [60]. This counterintuitive mechanism may be due to compensatory activation of other pathways, which may ultimately lead to an antidepressant effect. Further studies are needed to quantify its effect on these pathways and their downstream products to identify physiological outcomes and therapeutic relevance.

Taken together, the findings of this study suggest that aminophylline exhibits antidepressant-like effects in a chronic restraint stress model and may act through multiple neurobiological pathways implicated in depression. The observed behavioral improvements, including early reductions in immobility and efficacy at moderate doses within this model, warrant further investigation. However, these findings derive from a preclinical paradigm and should not be interpreted as demonstrating clinical advantage over established antidepressants, as differences in pharmacodynamics, dosing strategies, and human disease complexity cannot be fully recapitulated in rodents.

As a methylxanthine, aminophylline represents a pharmacologically interesting compound whose potential central effects merit additional study. Exploration of structurally related compounds such as theobromine and caffeine may also be informative, although comparative efficacy and mechanism remain to be determined.

Future studies employing targeted molecular and biochemical approaches, including quantitative PCR and western blotting to assess neurotrophic and inflammatory markers, corticosterone quantification to evaluate hypothalamic–pituitary–adrenal (HPA) axis activity, and immunohistochemistry to examine neurogenesis-related markers in stress-responsive brain regions, would be necessary to validate the predicted mechanistic pathways. In addition, RNA sequencing and targeted proteomic analyses could provide higher-resolution mapping of transcriptional and protein-level changes and help clarify whether the pathways identified in the current bioinformatic analyses are directly modulated by aminophylline under chronic stress conditions.

Several limitations should be acknowledged. The sequential exposure of mice to multiple behavioral assays may have introduced carryover effects. Furthermore, the molecular docking and transcriptomic analyses were exploratory and not validated in the same experimental cohort, and the gene expression dataset was derived from theophylline exposure rather than aminophylline administration within the present CRS model. Accordingly, mechanistic interpretations remain hypothesis-generating.

In summary, this study provides preliminary preclinical evidence that aminophylline attenuates depressive-like behaviors in a chronic stress paradigm. While the findings support further investigation, additional mechanistic validation and replication across models are required before conclusions regarding therapeutic repositioning can be established.

## Supplementary Information

Below is the link to the electronic supplementary material.

Supplementary Material 1

## Data Availability

The datasets generated and/or analyzed during the current study are available from the corresponding author upon reasonable request.

## Abbreviations

A05: Aminophylline 5 mg/kg
A10: Aminophylline 10 mg/kg
A20: Aminophylline 20 mg/kg
ADORA1: Adenosine A1 receptor
ANOVA: Analysis of variance
CRS: Chronic restraint stress
DEGs: Differentially expressed genes
ECM: Extracellular matrix
FDR: False discovery rate
FLX: Fluoxetine 10 mg/kg
FST: Forced swimming test
GEO: Gene expression omnibus
HPA: Hypothalamic-pituitary-adrenal
HSD: Honestly significant difference
IP: Intraperitoneal
JAK-STAT: Janus kinase/signal transducers and activators of transcription
KEGG: Kyoto encyclopedia of genes and genomes
MAPK: Mitogen-activated protein kinase
MDD: Major depressive disorder
PDB: Protein data bank
PDE: Phosphodiesterase
PI3K/AKT: Phosphatidylinositol 3-kinase/ protein kinase B
PPAR: Peroxisome proliferator-activated receptor
qPCR: Quantitative polymerase chain reaction
SDF: Structure-data file
SEM: Standard error of the mean
SERT: Serotonin transporter
SSRI: Selective serotonin reuptake inhibitors
SST: Sucrose splash test
TST: Tail suspension test
VEH: Vehicle

## References

[1] Association AP. Diagnostic and statistical manual of mental disorders, Fifth Ed., Text Revision (DSM-5-TR^®^). 2022.

[2] Cui L et al. Major depressive disorder: hypothesis, mechanism, prevention and treatment. Signal transduction and targeted therapy, 2024. 9(1): p. 30. 10.1038/s41392-024-01738-y PMC10853571 38331979

[3] Holmes SE, et al. Lower synaptic density is associated with depression severity and network alterations. Nat Commun. 2019;10(1):1529. 30948709 10.1038/s41467-019-09562-7 PMC6449365

[4] Zhou X, et al. Systematic review of management for treatment-resistant depression in adolescents. BMC Psychiatry. 2014;14(1):340. 25433401 10.1186/s12888-014-0340-6 PMC4254264

[5] Köhler-Forsberg O, Cusin C, Nierenberg AA. Evolving issues in the treatment of depression. JAMA. 2019;321(24):2401–2. 31125042 10.1001/jama.2019.4990

[6] Mohammad Sadeghi H, et al. Drug repurposing for the management of depression: where do we stand currently? Life. 2021;11(8):774. 34440518 10.3390/life11080774 PMC8398872

[7] Lesmana MHS et al. Genomic-analysis-oriented drug repurposing in the search for novel antidepressants. Biomedicines, 2022. 10(8): p. 1947. 10.3390/biomedicines10081947 PMC9405592 36009493

[8] Wang C et al. Reducing neuroinflammation in psychiatric disorders: Novel target of phosphodiesterase 4 (PDE4) and developing of the PDE4 inhibitors. Mech Neuroinflamm, 2017: pp. 3–23.

[9] Li Y-F, et al. Antidepressant-and anxiolytic-like effects of the phosphodiesterase-4 inhibitor rolipram on behavior depend on cyclic AMP response element binding protein-mediated neurogenesis in the hippocampus. Neuropsychopharmacology. 2009;34(11):2404–19. 19516250 10.1038/npp.2009.66 PMC2743762

[10] Colasanto M, Madigan S, Korczak DJ. Depression and inflammation among children and adolescents: A meta-analysis. J Affect Disord. 2020;277:940–8. 33065836 10.1016/j.jad.2020.09.025

[11] Enache D, Pariante CM, Mondelli V. Markers of central inflammation in major depressive disorder: a systematic review and meta-analysis of studies examining cerebrospinal fluid, positron emission tomography and post-mortem brain tissue. Brain Behav Immun. 2019;81:24–40. 31195092 10.1016/j.bbi.2019.06.015

[12] Percie du Sert N, et al. The ARRIVE guidelines 2.0: Updated guidelines for reporting animal research. J Cereb Blood Flow Metabolism. 2020;40(9):1769–77. 10.1177/0271678X20943823 PMC7430098 32663096

[13] Care, I.o.L.A.R.C.o. and U.o.L. Animals, Guide for the care and use of laboratory animals. 1986: US Department of Health and Human Services, Public Health Service, National ….

[14] Tadich T, Tarazona AM. Replacement, reduction and refinement: Ethical considerations in the current applications of the 3Rs. Handbook of Bioethical Decisions. Volume I: Decisions at the Bench, 2023: pp. 667–683.

[15] Powell TR, Fernandes C, Schalkwyk LC. Depression-related behavioral tests. Curr Protocols Mouse Biology. 2012;2(2):119–27. 10.1002/9780470942390.mo110176 26069008

[16] Sharma P, Sharma S, Singh D. Apigenin reverses behavioural impairments and cognitive decline in kindled mice via CREB-BDNF upregulation in the hippocampus. Nutr Neurosci. 2020;23(2):118–27. 29847220 10.1080/1028415X.2018.1478653

[17] Jayakumar S, et al. Effect of fluoxetine on the hippocampus of Wistar albino rats in cold restraint stress model. J Clin Diagn research: JCDR. 2017;11(6):AF01. 10.7860/JCDR/2017/26958.9953 PMC5535338 28764145

[18] Dulawa SC, et al. Effects of chronic fluoxetine in animal models of anxiety and depression. Neuropsychopharmacology. 2004;29(7):1321–30. 15085085 10.1038/sj.npp.1300433

[19] Hodes GE, Hill-Smith TE, Lucki I. Fluoxetine treatment induces dose dependent alterations in depression associated behavior and neural plasticity in female mice. Neurosci Lett. 2010;484(1):12–6. 20692322 10.1016/j.neulet.2010.07.084 PMC4623584

[20] Somekawa-Kondo T, et al. Aminophylline, administered at usual doses for rodents in pharmacological studies, induces hippocampal neuronal cell injury under low tidal volume hypoxic conditions in guinea-pigs. J Pharm Pharmacol. 2013;65(1):102–14. 23215693 10.1111/j.2042-7158.2012.01566.x

[21] Meng Z-Z, et al. Effect of xiaoyaosan decoction on learning and memory deficit in rats induced by chronic immobilization stress. Evidence-Based Complement Altern Med. 2013;2013(1):297154. 10.1155/2013/297154 PMC3891437 24459529

[22] Trofimiuk E, et al. Stress and ketamine, bimodal influence on cognitive functions. Behav Brain Res. 2019;360:354–64. 30562568 10.1016/j.bbr.2018.12.030

[23] Son H et al. A chronic immobilization stress protocol for inducing depression-like behavior in mice. J Visualized Experiments (JoVE), 2019(147): p. e59546. 10.3791/59546 31157777

[24] Botanas CJ, et al. R (–)-methoxetamine exerts rapid and sustained antidepressant effects and fewer behavioral side effects relative to S (+)-methoxetamine. Neuropharmacology. 2021;193:108619. 34023336 10.1016/j.neuropharm.2021.108619

[25] Botanas CJ, et al. Methoxetamine produces rapid and sustained antidepressant effects probably via glutamatergic and serotonergic mechanisms. Neuropharmacology. 2017;126:121–7. 28867363 10.1016/j.neuropharm.2017.08.038

[26] Kalueff AV, et al. Neurobiology of rodent self-grooming and its value for translational neuroscience. Nat Rev Neurosci. 2016;17(1):45–59. 26675822 10.1038/nrn.2015.8 PMC4840777

[27] Lefter R, et al. A new biological approach in generating an irritable bowel syndrome rat model-focusing on depression in sucrose splash test and body weight change. Romanian Biotehnological Lett. 2018;2:p2018.

[28] Forli S, et al. Computational protein–ligand docking and virtual drug screening with the AutoDock suite. Nat Protoc. 2016;11(5):905–19. 27077332 10.1038/nprot.2016.051 PMC4868550

[29] Scapin G, et al. Crystal structure of human phosphodiesterase 3B: atomic basis for substrate and inhibitor specificity. Biochemistry. 2004;43(20):6091–100. 15147193 10.1021/bi049868i

[30] Kranz M, et al. Identification of PDE4B Over 4D subtype-selective inhibitors revealing an unprecedented binding mode. Bioorg Med Chem. 2009;17(14):5336–41. 19525117 10.1016/j.bmc.2009.03.061

[31] Cheng RK, et al. Structures of human A1 and A2A adenosine receptors with xanthines reveal determinants of selectivity. Structure. 2017;25(8):1275–85. e4. 28712806 10.1016/j.str.2017.06.012

[32] Coleman JA, Green EM, Gouaux E. X-ray structures and mechanism of the human serotonin transporter. Nature. 2016;532(7599):334–9. 27049939 10.1038/nature17629 PMC4898786

[33] Berman HM, et al. The protein data bank. Nucleic Acids Res. 2000;28(1):235–42. 10592235 10.1093/nar/28.1.235 PMC102472

[34] Kim S, et al. PubChem 2025 update. Nucleic Acids Res. 2025;53(D1):D1516–25. 39558165 10.1093/nar/gkae1059 PMC11701573

[35] Wu Q, et al. COACH-D: improved protein–ligand binding sites prediction with refined ligand-binding poses through molecular docking. Nucleic Acids Res. 2018;46(W1):W438–42. 29846643 10.1093/nar/gky439 PMC6030866

[36] Eberhardt J, et al. AutoDock Vina 1.2. 0: new docking methods, expanded force field, and python bindings. J Chem Inf Model. 2021;61(8):3891–8. 34278794 10.1021/acs.jcim.1c00203 PMC10683950

[37] Coghlan H et al. Hepatic cellular stress response pathways exhibit species differences in basal and inducible activity. bioRxiv, 2025: p. 2025.09. 22.677731. 10.1093/toxsci/kfag061 PMC13387358 42210022

[38] Langfelder P, Horvath S. WGCNA: an R package for weighted correlation network analysis. BMC Bioinformatics. 2008;9(1):559. 19114008 10.1186/1471-2105-9-559 PMC2631488

[39] Ritchie ME, et al. limma powers differential expression analyses for RNA-sequencing and microarray studies. Nucleic Acids Res. 2015;43(7):e47–47. 25605792 10.1093/nar/gkv007 PMC4402510

[40] Szklarczyk D, et al. The STRING database in 2023: protein–protein association networks and functional enrichment analyses for any sequenced genome of interest. Nucleic Acids Res. 2023;51(D1):D638–46. 36370105 10.1093/nar/gkac1000 PMC9825434

[41] Shannon P, et al. Cytoscape: a software environment for integrated models of biomolecular interaction networks. Genome Res. 2003;13(11):2498–504. 14597658 10.1101/gr.1239303 PMC403769

[42] Chin C-H, et al. cytoHubba: identifying hub objects and sub-networks from complex interactome. BMC Syst Biol. 2014;8(Suppl 4):S11. 25521941 10.1186/1752-0509-8-S4-S11 PMC4290687

[43] Kolberg L, et al. g: Profiler—interoperable web service for functional enrichment analysis and gene identifier mapping (2023 update). Nucleic Acids Res. 2023;51(W1):W207–12. 37144459 10.1093/nar/gkad347 PMC10320099

[44] Team RC. R: A language and environment for statistical computing. R Foundation for Statistical Computing, Vienna, Austria 2016. https://www.R-project.org.

[45] Wickham H. Data analysis, in ggplot2: elegant graphics for data analysis. Springer; 2016. pp. 189–201.

[46] Sadeghi MA, et al. Phosphodiesterase inhibitors in psychiatric disorders. Psychopharmacology. 2023;240(6):1201–19. 37060470 10.1007/s00213-023-06361-3

[47] Hu Y, Yiu V, Clark R. Etiology of Depression: biological and environmental factors in the development of depression. J Student Res, 2021. 10(4).

[48] Remes O, Mendes JF, Templeton P. Biological, psychological, and social determinants of depression: a review of recent literature. Brain Sci. 2021;11(12):1633. 34942936 10.3390/brainsci11121633 PMC8699555

[49] Seewoo BJ et al. Validation of chronic restraint stress model in young adult rats for the study of depression using longitudinal multimodal MR imaging. Eneuro, 2020. 7(4). 10.1523/ENEURO.0113-20.2020 PMC7396811 32669346

[50] De Kloet ER, Joëls M, Holsboer F. Stress and the brain: from adaptation to disease. Nat Rev Neurosci. 2005;6(6):463–75. 15891777 10.1038/nrn1683

[51] Myers B, McKlveen JM, Herman JP. Glucocorticoid actions on synapses, circuits, and behavior: implications for the energetics of stress. Front Neuroendocr. 2014;35(2):180–96. 10.1016/j.yfrne.2013.12.003 PMC4422101 24361584

[52] Krystal JH, Kavalali ET, Monteggia LM. Ketamine and rapid antidepressant action: new treatments and novel synaptic signaling mechanisms. Neuropsychopharmacology. 2024;49(1):41–50. 37488280 10.1038/s41386-023-01629-w PMC10700627

[53] Chanaday NL, et al. The synaptic vesicle cycle revisited: new insights into the modes and mechanisms. J Neurosci. 2019;39(42):8209–16. 31619489 10.1523/JNEUROSCI.1158-19.2019 PMC6794917

[54] Dong X-x, Wang Y, Qin Z-h. Molecular mechanisms of excitotoxicity and their relevance to pathogenesis of neurodegenerative diseases. Acta Pharmacol Sin. 2009;30(4):379–87. 19343058 10.1038/aps.2009.24 PMC4002277

[55] Miller AH, Raison CL. The role of inflammation in depression: from evolutionary imperative to modern treatment target. Nat Rev Immunol. 2016;16(1):22–34. 26711676 10.1038/nri.2015.5 PMC5542678

[56] Halaris A, Sohl E, Whitham EA. Treatment-resistant depression revisited: a glimmer of hope. J personalized Med. 2021;11(2):155. 10.3390/jpm11020155 PMC7927134 33672126

[57] Zolezzi JM, et al. PPARs in the central nervous system: roles in neurodegeneration and neuroinflammation. Biol Rev. 2017;92(4):2046–69. 28220655 10.1111/brv.12320

[58] Menniti FS, Faraci WS, Schmidt CJ. Phosphodiesterases in the CNS: targets for drug development. Nat Rev Drug Discovery. 2006;5(8):660–70. 16883304 10.1038/nrd2058

[59] Kariharan T, et al. Central activation of PPAR-gamma ameliorates diabetes induced cognitive dysfunction and improves BDNF expression. Neurobiol Aging. 2015;36(3):1451–61. 25510319 10.1016/j.neurobiolaging.2014.09.028

[60] Duman RS, Voleti B. Signaling pathways underlying the pathophysiology and treatment of depression: novel mechanisms for rapid-acting agents. Trends Neurosci. 2012;35(1):47–56. 22217452 10.1016/j.tins.2011.11.004 PMC3278537

