# Supplementary material for: Potential antidepressant properties of aminophylline in male mice exposed to chronic restraint stress
Source: Pharmacol Rep. 2026 Apr 7;78(3):748–61. doi: 10.1007/s43440-026-00851-9 (PMC13275603; doi:10.1007/s43440-026-00851-9)
Supplement: Supplementary file 1 — Supplementary Material 1 [file 43440_2026_851_MOESM1_ESM.pdf]

Supplementary Table 1. Differential gene expression analysis. Summary of genes significantly altered in response to aminophylline treatment. A total of 221 genes were upregulated, and 392 genes were downregulated, highlighting potential molecular pathways involved in the drug's antidepressant-like effects.

Downregulated Genes			Upregulated Genes		
**Gene**	**LogFC**	**Adjusted P-value**	**Gene**	**LogFC**	**Adjusted P-value**
Kif2a	-0.27377	0.041697	Dbp	11.60003	8.45E-17
G6pd	-0.27507	0.036849	Serpini1	10.68126	7.72E-20
Prpf19	-0.28084	0.045822	Rims1	9.764981	9.50E-10
Nsf	-0.28205	0.024277	Cnksr2	9.706964	9.50E-10
Lrit1	-0.29082	0.031293	Asic3	9.134136	3.63E-10
Snn	-0.30109	0.044253	Sstr2	7.17897	3.13E-09
Akap11	-0.30397	0.036831	Ppp2r2c	6.153141	8.61E-11
Tp53	-0.3138	0.030733	Vom2r-ps45	4.81407	6.10E-08
P4ha1	-0.31931	0.044959	Myl3	4.769573	0.000321
Pitpna	-0.32123	0.026822	Axin1	4.121369	0.042654
Nbn	-0.32229	0.040475	Pbsn	3.97746	0.000148
Ruvbl1	-0.33313	0.005952	Casq2	3.887193	7.43E-06
Slc4a1	-0.33507	0.046529	Cd36-ps1	3.680741	3.78E-08
Akr1a1	-0.35025	0.017412	Klrb1c	3.663868	6.26E-06
Cyp2a2	-0.35082	0.049133	Pon1	3.663054	0.000466
Arf5	-0.35143	0.046136	Arg2	3.302566	0.000536
Chek2	-0.35513	0.021625	Uncx	3.134679	0.008614
Dkc1	-0.35645	0.016376	Pth	2.790296	0.000536
Pex3	-0.358	0.049064	Kap	2.634121	0.009094
C19h19orf53	-0.36123	0.0393	Neurod1	2.469799	0.020649
Plg	-0.36232	0.041413	Ube2d2b	2.425492	0.000739
Slc10a1	-0.36265	0.038454	Slc1a6	2.314579	0.025837
Grm7	-0.36273	0.024723	Twist2	2.248412	0.008614
Trpc1	-0.36355	0.027222	Sipa1l1	2.150319	0.002473
Clip2	-0.37242	0.025433	Slc6a4	2.149308	0.04871
Odc1	-0.37526	0.009628	Serpina10	2.111149	0.000933
Adcy3	-0.37687	0.034422	Tff3	1.877347	0.040504
Prkaa2	-0.38453	0.021625	Hao2	1.827148	0.033319

Aacs	-0.38676	0.008641	Mrgprf	1.801703	0.027213
Stau1	-0.38711	0.008828	Pkib	1.790165	0.019432
Capn9	-0.38714	0.030289	Homer3	1.736894	0.004919
Gsk3b	-0.39355	0.04101	Comp	1.703513	0.048167
Bag6	-0.39572	0.00344	Itga7	1.678032	0.00719
Stat1	-0.39868	0.028811	Lipc	1.674724	0.025837
Agxt2	-0.40164	0.019188	Cd244l1	1.639577	0.000108
Sharpin	-0.40327	0.037109	Gnmt	1.614523	0.025837
Mpg	-0.41051	0.026822	Ccl20	1.609066	0.016241
Gne	-0.41804	0.010334	Gdf10	1.589541	0.028875
Rtn4	-0.41887	0.006564	Cpt1b	1.563324	0.006515
Atp5f1c	-0.42159	0.017797	Sycp3	1.542433	0.028908
Hpn	-0.4225	0.009907	Ifng	1.526635	0.006515
Negr1	-0.42523	0.01514	Barhl1	1.522108	0.004919
Anxa5	-0.43146	0.025652	Hspb7	1.480943	0.049779
Naa35	-0.43577	0.017957	Dusp5	1.442846	0.032779
S100b	-0.43725	0.031593	Slc6a3	1.416617	0.025137
Sp4	-0.43862	0.031293	Rdh16	1.399306	0.00053
Ampd3	-0.44382	0.046961	Omd	1.374033	0.002473
Ywhae	-0.44554	0.006675	Fbp1	1.371887	0.001769
Bmpr1a	-0.44739	0.033812	Pdk4	1.36895	0.007042
Jag1	-0.45526	0.030686	Syt2	1.349643	0.021022
Mgat1	-0.45888	0.015464	Lrp2	1.333168	0.021939
Atp5f1a	-0.46093	0.010004	Snrk	1.304713	0.004198
Cdc123	-0.46269	0.016553	Stc1	1.300332	0.038415
Ywhag	-0.46472	0.017957	Akap12	1.238443	0.015093
Syk	-0.46522	0.017936	Cd5	1.2134	0.029499
Ppia	-0.46633	0.004288	Samd13	1.209227	0.02567
Prpsap2	-0.46985	0.020418	Slc4a7	1.202913	0.000944
Atp5f1d	-0.46988	0.013979	Il2ra	1.181366	0.025137
Fam162a	-0.47126	0.00871	Ctbp2	1.160261	0.002587
Thop1	-0.47235	0.015893	Nt5e	1.150964	0.002256
Abtb2	-0.4727	0.023953	Kcnj12	1.122981	0.039703
Hrk	-0.47862	0.024064	Mia	1.104665	0.009628

Xylt1	-0.4805	0.003597	Arfip1	1.096171	0.000536
Tox4	-0.48464	0.012981	Cbs	1.055189	0.006022
Rxra	-0.48987	0.033812	Nid1	1.046925	0.004681
Scg3	-0.49003	0.034647	Slc19a1	1.043483	0.002473
Acox1	-0.49052	0.008131	Mapk12	1.034419	0.039918
Nfyb	-0.49345	0.039703	Cnn3	1.025398	4.21E-05
Fcgr2b	-0.49411	0.029389	Ajuba	1.022388	0.001482
Grm3	-0.49718	0.033282	Syt4	1.008142	0.000137
Bmp15	-0.50376	0.046294	Stk10	1.005256	0.002114
Ppm1f	-0.50977	0.002575	Ret	1.001764	0.034422
Fuca1	-0.51186	0.043699	Flt4	0.988165	0.000563
Prps2	-0.51889	0.033319	Slc2a4	0.977029	0.009946
Ndufv2	-0.52041	0.000502	Avpr1a	0.958283	0.004896
Cxxc4	-0.52046	0.005952	Mpp3	0.904256	0.04817
Ecel1	-0.52224	0.007364	Abcd2	0.88734	8.97E-05
Vamp2	-0.52497	0.03098	Ccn5	0.886798	0.028908
Erp29	-0.52576	0.045593	Lmnb1	0.870171	0.021144
Pfkl	-0.52744	0.014375	Fcnb	0.836149	0.030444
Dlgap1	-0.52926	0.016553	Pparg	0.836135	0.014849
Podxl	-0.53174	0.024064	Tsc1	0.832309	0.031593
Ucp3	-0.53653	0.031593	Cdc25b	0.828543	0.001161
Mtr	-0.54233	0.006196	Igd	0.824997	0.010031
Clta	-0.54279	0.005659	Gcgr	0.80312	0.041617
Mapk14	-0.54378	0.001154	Soat1	0.796193	0.034784
Entpd2	-0.54412	0.014702	Gcm1	0.795593	0.013897
Mtpn	-0.54825	0.046294	Htr4	0.793348	0.019368
Serpina3n	-0.54869	0.006515	Stk3	0.790325	0.001528
Pik3c2g	-0.54999	0.039363	Furin	0.777804	0.00023
Tg	-0.55212	0.022503	Slc9a5	0.775968	0.000228
Tsc2	-0.55422	0.002497	Ppfia4	0.773295	0.025837
Cyp3a2	-0.55673	0.006515	Cpt2	0.761535	0.001165
Neu2	-0.55717	0.033812	Alox15	0.75472	0.029389
Tsc22d1	-0.55775	0.008731	Maf	0.752028	0.009225
Slc28a1	-0.56239	0.003125	Oplah	0.749898	0.019432

Taf9b	-0.56406	0.025837	Slc6a1	0.746583	0.000704
Septin9	-0.56735	0.027222	Hr	0.740172	0.031593
Arpc1a	-0.57176	0.020231	Abcc1	0.739792	0.001586
Cma1	-0.57219	0.034647	Aqp5	0.728856	0.030218
Ackr2	-0.57302	0.033379	Defb4	0.726807	0.000716
Arl4a	-0.57457	0.019741	Fbln5	0.723847	0.012539
Abcc2	-0.58323	0.008641	Atp5mc3	0.714549	0.000706
Marcksl1	-0.58554	0.007695	Lifr	0.711955	0.024064
Faim2	-0.5862	0.005659	Gp5	0.709214	0.023825
Bard1	-0.58946	0.045593	Fgf9	0.704161	0.030104
Atp5mc1	-0.59037	0.000466	Cblb	0.69466	0.001515
Ncoa3	-0.59122	0.002101	Smc3	0.68808	0.000472
Brca1	-0.59264	0.028811	Inhbe	0.683734	0.010119
Pde4b	-0.59421	0.000921	Plce1	0.681708	0.018116
Ptgfrn	-0.60359	0.010996	Runx1	0.680041	0.014331
Rho	-0.60472	0.035998	Lypd3	0.674013	0.031293
Fgf2	-0.60626	0.002264	Pdgfd	0.655665	0.033319
Kcnma1	-0.60797	0.010287	Crot	0.654775	0.007273
Syt11	-0.61529	0.027222	Slc12a5	0.647754	0.000435
Slc25a21	-0.62285	0.005748	Map3k12	0.645498	0.005952
Ctsl	-0.62708	0.04101	Sort1	0.644116	0.005484
Celsr2	-0.62793	0.046663	Limk1	0.643539	0.004896
Serpind1	-0.62883	0.00061	Rfc2	0.641221	0.007325
Gabrr2	-0.63022	0.0042	Ptges	0.639646	0.021239
Apobec1	-0.63157	0.016456	Gusb	0.636505	0.005403
Vipr1	-0.63168	0.003962	Ebf1	0.63627	0.047446
Id3	-0.63352	0.003042	Herpud1	0.633002	0.000867
Map2k5	-0.63599	0.001208	Arl5a	0.623142	0.001114
Acsm2	-0.63697	0.012542	Plcd4	0.61986	0.045968
Ptpn7	-0.6375	0.001454	Lama1	0.619594	0.009797
Tk1	-0.63892	0.003393	Sycp2	0.617852	0.004359
Slc6a18	-0.64061	0.001543	Nfe2l2	0.616141	0.02992
Tmbim6	-0.64126	0.034482	Cyp2b3	0.614451	0.048079
Men1	-0.64279	0.001165	Smo	0.613679	0.010192

Rab3a	-0.64448	0.049768	Atrn	0.613164	0.015346
Ifit1	-0.64533	0.028103	Srd5a1	0.612525	0.003072
P2ry2	-0.64534	0.030218	Adamts1	0.611226	0.025799
Dnajc3	-0.64887	9.05E-05	Klhl41	0.610846	0.032913
Gclm	-0.65034	0.000255	Zdhhc2	0.604595	0.003988
Rpl32	-0.65106	0.048302	Slc23a2	0.602654	0.02992
Sh3gl3	-0.65384	0.016257	Nr1h3	0.595646	0.038494
Pebp1	-0.65627	0.001541	Nlgn2	0.593973	0.002611
Wee1	-0.65792	0.001767	Tmpo	0.593564	0.025793
Slc2a2	-0.66214	0.008614	Aox1	0.591082	0.016279
Kras	-0.66234	0.011819	Gng5	0.589996	0.009435
Gcg	-0.66552	0.033944	Polb	0.589244	0.008666
Clec4f	-0.66802	0.034811	Cdk5rap2	0.588402	0.007687
Lat	-0.66921	0.033933	Ngb	0.586736	0.008306
Ddx39a	-0.67121	0.000569	Gemin2	0.586433	0.002264
Dio1	-0.67187	0.002974	Slc28a2	0.585654	0.033881
Mylk2	-0.67252	0.004916	Pten	0.575646	0.013708
Tas1r2	-0.67326	0.010004	Bhlhe40	0.571973	0.026171
Cavin3	-0.67333	0.025793	Zfp384	0.569847	0.003072
Iapp	-0.67864	0.036849	Slc38a2	0.56598	0.008966
Pdk1	-0.68316	0.010004	Fstl1	0.563306	0.046401
Pola2	-0.6851	0.036001	Ppara	0.555204	0.005837
Slc13a2	-0.69272	0.048601	Prkdc	0.552405	0.008306
Ppp1ca	-0.69588	0.00282	Eif5	0.549783	0.006515
Ssb	-0.69755	0.00871	Rsad2	0.545076	0.039918
Ube2g1	-0.69933	0.000293	Slc32a1	0.538985	0.041581
Kcnb1	-0.69977	0.043528	Nup107	0.532044	0.001722
Serpina3m	-0.70015	0.003649	Cyp7b1	0.52968	0.021939
Gjb4	-0.70276	0.001469	Grem1	0.516663	0.015497
Fabp7	-0.70539	0.016553	Plcl1	0.513951	0.019188
Timp2	-0.70761	0.008614	Msh2	0.51121	0.005298
Rgs7	-0.70897	0.010004	Ica1	0.500094	0.001801
C5ar1	-0.71207	0.031593	Trim3	0.499833	0.043956
Hsd3b5	-0.7122	0.001451	Arnt	0.494451	0.021939

Slc9a2	-0.71383	0.009861	Txnip	0.494	0.034348
Gnb1	-0.71446	0.001292	Lamb2	0.489166	0.042569
Musk	-0.71473	0.004359	Dlat	0.486816	0.00361
Fas	-0.71537	0.033944	Fntb	0.486349	0.026171
Pabpc1	-0.71725	0.000656	Mapk9	0.484643	0.019921
Gabra3	-0.71996	0.026869	Hnrnpk	0.47404	0.001386
Kcna5	-0.72162	0.023953	Mtdh	0.473912	0.003925
Lyz2	-0.72401	0.007307	Scn1a	0.469196	0.027189
Cacnb3	-0.72572	0.006022	Adrb2	0.469169	0.020056
Ppp1r9a	-0.72799	0.025793	P3h1	0.467636	0.046593
Crx	-0.72845	0.000536	Zfp709	0.462167	0.031593
Gpr12	-0.73015	0.000452	Gls2	0.461465	0.00968
Olr1	-0.73073	0.017121	Lrrn3	0.456056	0.017031
Akr1d1	-0.73481	0.028972	Abcc3	0.45584	0.034348
Rab12	-0.73521	0.005232	Shank3	0.455742	0.021039
Drd4	-0.73541	0.019921	Pmp22	0.449687	0.04007
Mef2d	-0.7444	0.001344	Scamp5	0.449556	0.048019
Gna15	-0.74507	0.002473	Rnf112	0.448405	0.033812
Trpv6	-0.74607	0.006515	Nop58	0.448189	0.003393
Nr0b1	-0.7496	0.00023	Cntf	0.441867	0.034498
Psen1	-0.7497	0.004241	Pfkfb4	0.438595	0.009616
Ccr3	-0.74981	0.00361	Plcg2	0.438547	0.031081
Ces2e	-0.75076	0.001528	Pex12	0.437677	0.016279
Dclk1	-0.76367	0.001344	Adgrf5	0.436405	0.025764
Nell2	-0.76441	0.000466	Ahcy	0.435073	0.008306
Thra	-0.76567	0.030444	Akap8	0.432247	0.028276
Csde1	-0.76648	0.006099	Per2	0.427163	0.035298
Slk	-0.76716	0.008641	Cast	0.424532	0.005298
Gad2	-0.76859	0.002339	Tcp1	0.422562	0.003813
Cox17	-0.77265	0.000536	Kidins220	0.404254	0.013078
Ucp1	-0.77601	0.001651	Vps33b	0.399972	0.007695
Rock2	-0.77647	0.001149	Tmod1	0.398122	0.046533
Haao	-0.77809	0.000704	Stk39	0.397569	0.046529
Cyp2c6v1	-0.78317	0.008805	Fez1	0.39482	0.028508

Dusp6	-0.7853	0.009737	Ptpn21	0.389583	0.045919
Cacna1i	-0.78607	0.034811	Bhlhe41	0.386009	0.03127
Esr2	-0.78991	0.018044	Bckdhb	0.379055	0.009339
Nsmf	-0.7908	0.026171	Gpr37l1	0.371981	0.046629
S100a10	-0.79235	0.001344	Adcy4	0.371439	0.044905
Ampd1	-0.79852	0.002339	Pglyrp1	0.36219	0.044051
Il6	-0.80027	0.000448	Acsl3	0.3602	0.008966
Csf3	-0.80543	0.024849	Rnpep	0.356448	0.0112
Hgs	-0.81113	0.009573	Timm17a	0.350132	0.0115
Mpp2	-0.81169	0.008842	Npm1	0.336566	0.026822
Map1b	-0.81189	0.000608	Hacl1	0.334849	0.022909
Kcnc2	-0.81212	0.008614	Impa1	0.330708	0.007421
Ltb4r	-0.81348	0.019579	Cltc	0.327966	0.033933
Cyp2c22	-0.81428	0.000293	Ppp1r15a	0.322827	0.008641
Sycn	-0.81649	0.031957	Slc7a5	0.318781	0.033846
Rasgrp1	-0.82003	0.034368	Plcg1	0.318621	0.024549
Cacna1e	-0.82014	0.000656	Fen1	0.309089	0.040475
Gsk3a	-0.82277	0.006022	Ap3m1	0.299717	0.046174
Hsd17b10	-0.82547	0.000306	Pitpnb	0.28915	0.034854
Nfic	-0.82654	0.023189	Arrb2	0.284338	0.010413
Adra1d	-0.83118	0.001474	Dync1i2	0.27493	0.013302
Nme1	-0.83238	0.018044	Prps1	0.268858	0.032694
Fosl1	-0.83558	0.011756	Map4k3	0.216976	0.046964
Glrx3	-0.83581	0.000775			
Aanat	-0.83655	0.00265			
Myc	-0.83669	0.005439			
Kcna2	-0.83988	0.002075			
Anpep	-0.84363	0.016611			
Npy1r	-0.84568	0.000706			
St8sia3	-0.84679	0.003393			
Cryab	-0.84854	0.038994			
Gabrr3	-0.84976	0.034967			
Amacr	-0.85188	0.010172			
Dao	-0.85286	0.017684			

Cyp1a1	-0.85661	0.020649
Nudt1	-0.86079	0.001928
Rph3a	-0.86722	0.000787
Map6	-0.86867	0.009602
Ppp3ca	-0.86874	0.032779
Mecp2	-0.87101	0.000448
Ybx1	-0.87319	0.000302
Pts	-0.87433	0.021144
Cplx2	-0.87448	0.004275
Tnfrsf8	-0.87627	2.01E-05
Tat	-0.88268	0.000435
Bpifa2	-0.8856	0.000536
Prss8	-0.88589	0.015093
Smad2	-0.88632	0.012105
Pcyt1a	-0.88919	0.016553
Sag	-0.88949	0.02132
Bdkrb1	-0.88982	0.001172
Lep	-0.89043	0.011801
Cxcl12	-0.89046	0.002114
Cckar	-0.89165	0.008805
Rpl29	-0.89213	0.00966
C3	-0.89325	0.008731
Cacng1	-0.90203	0.007514
Abcb11	-0.90412	0.000244
Gfra3	-0.90463	0.009737
S1pr2	-0.90605	0.001715
Txn2	-0.90957	0.02992
Rxrg	-0.91056	0.019963
Met	-0.91172	3.32E-05
Grp	-0.91227	0.021022
Kcng1	-0.91457	0.027626
Hes1	-0.91551	0.000435
Hrh3	-0.93064	0.033944

Myo9a	-0.93322	0.000415
C4bpa	-0.93424	0.000198
Sds	-0.93472	0.008306
Kcnk9	-0.93881	0.00968
Srcin1	-0.94118	0.000162
LOC134479803	-0.94292	0.034046
Mat1a	-0.9481	0.017465
Cyp2f4	-0.95335	0.003962
Itga1	-0.95568	0.008731
Vdr	-0.95672	0.000242
Mafb	-0.96013	0.003042
Epo	-0.96535	0.007346
Egr4	-0.96729	0.033944
Ccr5	-0.96971	0.00889
Slc18a3	-0.96974	0.039363
Hemgn	-0.97295	0.000536
Lilrb2	-0.97389	0.000108
Cul5	-0.97653	0.003757
Slc25a4	-0.97812	6.38E-05
Cpa3	-0.98277	0.035879
Psma3	-0.98391	0.0042
Zp3	-0.98403	0.000285
Gucy1a1	-0.98823	0.033933
Ran	-0.99225	7.88E-05
Rps7	-0.99474	0.039703
Ltbp1	-0.99519	0.006727
Foxm1	-0.9978	0.000748
Gata1	-1.00704	0.002831
Cyp3a23-3a1	-1.01128	5.48E-05
Rab15	-1.02826	0.014315
Rab26	-1.02923	0.020163
Chrna1	-1.04116	3.28E-05
Adora1	-1.0416	0.005952

Id2	-1.04791	0.006515
Adra2b	-1.05154	8.01E-06
Cdk4	-1.05466	0.002264
Adra1b	-1.06748	0.006022
Slc7a7	-1.07301	0.004198
Vch	-1.07637	0.003292
Actg2	-1.07643	0.004681
Adrb3	-1.09002	0.047446
Nos1ap	-1.09123	0.002075
F2r	-1.09217	0.014849
Atp7b	-1.09966	0.003371
Glra3	-1.10094	0.001069
Nap1l1	-1.10108	0.000306
Myo5b	-1.10969	0.030403
Vom1r101	-1.11005	0.001273
Slc17a7	-1.11118	0.001161
Npy4r	-1.11559	0.000448
Hmgb1	-1.11751	0.00013
Kdr	-1.11987	0.026466
Atp5me	-1.12099	0.0046
Ibsp	-1.12113	0.00013
Ghrl	-1.12135	0.005013
Rest	-1.12972	0.0046
Gss	-1.1315	0.004681
Thpol1	-1.13166	0.001586
Hal	-1.13415	0.000226
Slc5a1	-1.13642	0.000306
P2rx2	-1.14295	0.003072
Slc10a2	-1.15228	0.033812
Galr1	-1.15267	0.000609
Hnrnpu	-1.15286	0.003888
Gdf15	-1.16046	0.001208
Tomm20	-1.16974	2.17E-05

Pira2	-1.17231	0.00954
Ar	-1.17405	0.017029
Ccng1	-1.18939	2.51E-05
Cd8a	-1.20125	0.004008
Sst	-1.2046	0.006196
Arf3	-1.20502	0.007687
Vapa	-1.22224	0.000387
Psmb4	-1.26277	0.000118
Cdh6	-1.27685	0.026096
Il2rb	-1.2948	0.000173
Calcr	-1.30294	0.007134
Tubb2a	-1.30833	0.006022
Ryr2	-1.31588	0.000739
Htr1f	-1.31749	2.58E-05
Slc7a9	-1.31846	0.001161
Eloc	-1.32743	0.000569
Lrrc7	-1.34423	0.019921
Grm5	-1.43692	0.009946
Hes5	-1.45638	0.006367
Clock	-1.47813	0.000631
Grpr	-1.48613	0.000452
Hpcal4	-1.51584	0.007695
Tbxas1	-1.52078	0.00163
Slc22a25	-1.53724	0.001292
Hoxa1	-1.54402	0.010188
Akap5	-1.54965	0.001528
Or7a38c	-1.54971	2.88E-06
Gria1	-1.63657	3.83E-07
Rpl6	-1.66506	0.034426
LOC120099768	-1.74889	0.0042
Tpsb2	-1.77132	0.001589
Pigr	-1.80987	0.000428
Hsd17b2	-1.86468	0.000314

Spam1	-1.87987	0.001528
Gucy2c	-1.99677	0.017797
Slc26a5	-2.26613	0.007364
Retnla	-2.4206	0.000536
Slc14a1	-2.6406	0.033319
Mybph	-2.69224	1.62E-06
Cyp2a3	-2.7412	0.001289
Cyp2e1	-2.9957	0.014566
Glra1	-3.01205	5.82E-09
Cyp1a2	-3.01805	0.00344
Tsga10	-3.20851	3.28E-05
Acly	-3.52224	0.034498
Serpina3c	-3.63952	0.04429
Cd1d1	-3.67459	0.028336
Apoc3	-3.8749	0.019877
Myl11	-3.92961	0.000306
Alb	-4.10086	0.034422
Krt1	-4.25237	3.63E-05
Cebpg	-4.6826	0.004413
Coro1b	-4.75506	0.011082
Pah	-5.02227	0.000152
Drd5	-5.24953	1.43E-13
Sctr	-5.41643	8.22E-05
Hp	-5.91572	0.000285
Glra2	-6.48894	3.51E-05
Cd40	-6.91279	6.65E-15
Gpr85	-9.18252	1.04E-16
Ptgds	-10.5926	0.000306
